# Knowledge, Attitudes, and Practices Regarding Antimalarial Chemoprophylaxis in U.S. Peace Corps Volunteers — Africa, 2013

**Published:** 2014-06-13

**Authors:** Keren Z. Landman, Kathrine R. Tan, Paul M. Arguin

**Affiliations:** 1 EIS officer, CDC; 2 Malaria Branch, Division of Parasitic Diseases and Malaria, Center for Global Health, CDC

Long-term travelers to areas where malaria is endemic are at risk for this potentially fatal disease; however, malaria can be prevented through the use of insecticide-treated bednets, mosquito repellents, and chemoprophylaxis. Three options for chemoprophylaxis are available in the Africa region: mefloquine, doxycycline, and atovaquone-proguanil. These options differ by dosing regimen, cost, and side effect profile (1) (Table). Long-term adverse effects of these drugs have been reported rarely.

Peace Corps volunteers (PCVs) are among the long-term travelers for whom chemoprophylaxis is often recommended. The U.S. Peace Corps provides comprehensive health care to PCVs, including chemoprophylaxis to PCVs serving where malaria is endemic, and works to continually improve PCVs’ understanding of health risks and the risks and benefits of antimalaria chemoprophylaxis. PCV adherence to malaria chemoprophylaxis is required by Peace Corps policy, and nonadherence can lead to termination from Peace Corps service (2). Peace Corps medical officers (PCMOs) are nonvolunteer health-care workers who provide primary care to PCVs. Because of concern about increasing numbers of cases of severe malaria among PCVs, CDC investigated PCVs’ and PCMOs’ knowledge, attitudes, and practices regarding chemoprophylaxis to develop recommendations to improve adherence.

During August 19–September 30, 2013, anonymized Internet surveys assessing knowledge about, experience with, and perceptions of malaria and chemoprophylaxis were administered to PCVs and PCMOs serving in 18 African countries where antimalarial chemoprophylaxis is uniformly recommended. PCVs reporting taking daily medications at any time each day or taking weekly medications with no more than 8 days between doses were defined as adherent. Survey data were analyzed to identify opportunities for program improvement.

Responses were received from 974 PCVs and 47 PCMOs, yielding response rates of 42% and 90%, respectively. A total of 447 (47%) PCVs reported being prescribed mefloquine, whereas 391 (41%) were prescribed doxycycline, and 120 (13%) were prescribed atovaquone-proguanil. Adherence was reported by 612 (73%) PCVs and was highest among those prescribed atovaquone-proguanil (92 [88%]); 277 (82%) of those prescribed doxycycline and 243 (62%) of those prescribed mefloquine reported adherence. The most common reasons for nonadherence were forgetting to take chemoprophylaxis (576 [90%]), usually during in-country travel (287 [50%]); fear of long-term adverse effects (349 [54%]); and current adverse effects (324 [51%]). A total of 228 (23%) PCVs were not worried about malaria, mostly because they believed malaria was a minor illness (192 [84%]). Twenty-six (76%) of responding PCMOs indicated Peace Corps policy regarding chemoprophylaxis improved their ability to prevent malaria, and 44 (94%) appropriately indicated that known side effects were important reasons for changing chemoprophylaxis.

The findings in this report are subject to at least four limitations. First, the response rate among PCVs was only 42%, and nonresponse bias might have affected the results. For example, PCVs without strong feelings about chemoprophylaxis or preventive medicine might have been less likely to participate. Second, because the survey was administered using the Internet, selection bias might have occurred (e.g., selecting for PCVs in urban areas or those with Internet-enabled mobile devices). Third, because PCVs knew they were expected to be adherent to chemoprophylaxis, social desirability bias might have occurred. Finally, the exclusion of respondents from countries with geographically heterogeneous recommendations regarding chemoprophylaxis might have selected for PCVs exposed to fewer conflicting recommendations about the need for chemoprophylaxis.

In this survey, chemoprophylaxis adherence was reported by 73% of PCVs, and 54% and 51% of nonadherent PCVs reported fears of long-term adverse effects and current side effects, respectively. PCMOs recognized that chemoprophylaxis should be changed for known side effects. Changing chemoprophylaxis because of side effects might improve adherence, and the Peace Corps has already made changes to its policy that simplify this process and strengthen PCVs’ and PCMOs’ malaria education. For example, in December 2012, policy was changed from recommending mefloquine as first-line chemoprophylaxis to recommending all options equally. Additionally, the training curriculum delivered by PCMOs to PCVs has been updated and standardized (Barry G. Simon, MD, Peace Corps, personal communication, August 2013). Further interventions might include reminders for PCVs, additional education about chemoprophylaxis safety and malaria risk, and continued support for improved PCV-PCMO communication.

Since 1961, seven PCVs have died from malaria infections acquired during Peace Corps service, and malaria case rates among PCVs have reached as high as 18 cases per 100 volunteer years during the period 2009–2012 (3). CDC has previously assisted Peace Corps by serving as a reference laboratory for the diagnosis of malaria by blood smear and the diagnosis of mefloquine prophylaxis failure by determining blood mefloquine levels (4). CDC continues to work with Peace Corps toward improving PCV knowledge about and adherence to malaria chemoprophylaxis.

## Figures and Tables

**TABLE:** Drugs for malaria chemoprophylaxis in travelers to Africa

Prophylaxis	Dosing	Cost per month*	Side effect profile
Mefloquine	Weekly	$53	Neuropsychiatric
Doxycycline	Daily (with food)	$32	Skin/gastrointestinal, vaginal candidiasis
Atovaquone-proguanil	Daily	$236	Few side effects

* Source: Adachi K, Coleman MS, Khan N, et al. Economics of malaria prevention in US travelers to West Africa. Clin Infect Dis 2014;58:11–21.
